# The complete chloroplast genome sequence of cultivated *Prunus persica* cv. ‘Sovetskiy’

**DOI:** 10.1080/23802359.2021.1972861

**Published:** 2021-09-09

**Authors:** Fedor Sharko, Maria Gladysheva-Azgari, Svetlana Tsygankova, Irina Mitrofanova, Eugenia Boulygina, Natalia Slobodova, Anatoliy Smykov, Sergey Rastorguev, Artem Nedoluzhko

**Affiliations:** a National Research Center “Kurchatov Institute”, Moscow, Russia; b Nikita Botanical Gardens – National Scientific Centre of the Russian Academy of Sciences, Yalta, Russia; c Faculty of Biosciences and Aquaculture, Nord University, Bodø, Norway

**Keywords:** Chloroplast genome, peach, Rosaceae, annotation, nanopore sequencing

## Abstract

The peach (*Prunus persica* L. Batsch) is one of the important stone fruit crops in the Crimea Peninsula and the southern part of Russia. The complete chloroplast genome of the peach cultivar ‘Sovetskiy’ is published in this paper. The chloroplast genome size is 157,756 bp. It contains 126 genes, including 81 protein-coding genes (PCGs), eight ribosomal RNA (rRNA) genes, and 37 transfer RNA (tRNA) genes. The chloroplast genome also contains a large single-copy region of 85,960 bp, a small single-copy (SSC) region of 19,045 bp, and two inverted repeats regions of 26,375 bp and 26,372 bp. The overall base composition of the genome in descending order is 31.2% – A, 32.1% – T, 18.7% – C, and 18.0% – G. The total GC content of the chloroplast genome is 36.7%. Maximum-likelihood phylogenetic analysis involving nine chloroplast genomes of the *Prunus* genus revealed a separate cluster for *P. persica* and its possible landrace – *P. ferganensis*.

The peach (*Prunus persica* L. Batsch) is a species of the Rosaceae family (Bielenberg et al. 2009). This perennial stone fruit tree was domesticated at least 7500 years ago in China (Zheng et al. 2014). Peach tree is widely cultivated worldwide in continental or temperate climates (Li et al. 2019). The natural conditions of the Crimea Peninsula are favorable for peach horticulture development. Nikita Botanical Garden has one of the largest peach germplasm collections which contain more than 900 cultivars and forms. Moreover, new cultivars with important agricultural traits are actively created by the local breeders (Smykov 2019). In this paper, we present a complete assembly of the chloroplast genome of the peach cultivar *Prunus persica* cv. ‘Sovetskiy’ (Yezhov et al. 2005), which is known as one of the first cultivars created in the Soviet Union by crossing the ‘Golden Jubilee’ and ‘Narindzhi Pozdny’ cultivars. The peach cultivar ‘Sovetskiy’ was bred by Ivan Ryabov in the early 1950s and belongs to the Iranian peach cultivar group (Yezhov et al. 2005).

Genomic DNA was extracted from the ‘Sovetskiy’ cultivar (Nikita Botanical Garden, collection number − 40) leaves following the modified methodology described previously (Lo Piccolo et al. 2012). The Rapid Sequencing Kit (SQK-RAD004) and GridION genome analyzer (Oxford Nanopore Technologies, Kidlington, UK) were used for the genomic DNA sequencing.

535,545 GridION long reads (average length: 34.16 kbp) were generated for ‘Sovetskiy’ cultivar peach specimen. Obtained reads were mapped to the *P. persica* chloroplast reference genome (NC_014697.1) using minimap2 (Li 2018) with default parameters (coverage was 2185 X). Only 979,641 chloroplast genome reads were used for subsequent *de novo* assembly.

*De novo* assembly of the *P. persica* cv. ‘Sovetskiy’ chloroplast genome was performed using the Flye program (Kolmogorov et al. 2019) with polisher iterations. The chloroplast genome was annotated by using the CHloe web service (https://chloe.plantenergy.edu.au/).

The chloroplast genome of *P. persica* cv. ‘Sovetskiy’ (GenBank MZ065355) is 157,756 bp in length and has a circular structure. Chloroplast genome contains 126 genes, including − 81 protein-coding gene (PCG), eight ribosomal RNA (rRNA) genes, and 37 transfer RNA (tRNA) genes. Among these genes, 16 genes (*ndh*B, *pet*B, *pet*D, *rpl2*, *trn*A-UGC, *trn*G-UCC, *trn*I-GAU, *trn*L-UAA, *atp*F, *ndh*A, *rpl16*, *rpo*C1, *rps12*A, *rps16*, *trn*K-UUU, *trn*V-UAC) have one intron and three genes (*ycf3*, *rps12*B, *clp*P1) have two introns. In this genome, eight PCG (*ndh*B, *rpl2*, *rpl23*, *rps12*B, *rps19*, *rps7*, *ycf1*, and *ycf2*), four rRNA genes (*rrn16*, *rrn23*, *rrn4.5*, and *rrn5*), and seven tRNA genes (*trn*A-UGC, *trn*I-CAU, *trn*I-GAU, *trn*L-CAA, *trn*N-GUU, *trn*R-ACG, and *trn*V-GAC) are duplicated, the others genes have a single copy.

The complete chloroplast genome sequences of eight species of the *Prunus* genus were aligned with each other using the MUSCLE v3.8.1551 program (Edgar 2004) and then were used to construct phylogenic maximum-likelihood (ML) tree using the RaxML software v8.0.0 (Stamatakis et al. 2008) with 1000 bootstrap parameter. The complete chloroplast genome of almond (*P. amygdalus*) (KY085904.1) was used as an outgroup. Phylogenetic analysis based on the whole chloroplast sequences of *Prunus* species presents ‘traditional’ topology (Figure 1) which has been previously described. Cultivar ‘Sovetskiy’ was clustered with the cultivar ‘Nemared’ (NC014697.1) and Fergana peach – *P. ferganensis* (MK798146.1) which is usually considered as a landrace of *P. persica* (Verde et al. 2013; Xin et al. 2021).

**Figure 1.**
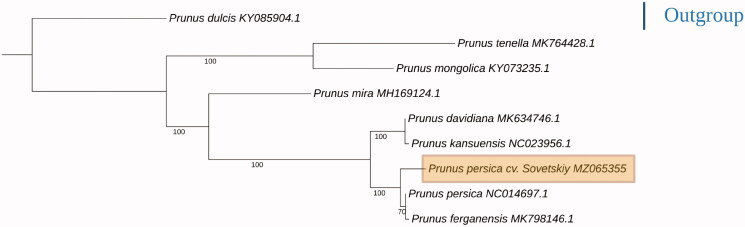
Phylogenic maximum-likelihood tree based on *Prunus* chloroplast genomes.

This study represents the first chloroplast genome assembly and annotation of cultivated *P. persica* cv. ‘Sovetskiy’. These data will be a potential genetic resource for peach cultivar identification and breeding.

## Data Availability

A specimen is grown at Nikita Botanical Garden (http://nbgnsc.ru/en, contact person: Anatoliy Smykov – e-mail: selectfruit@yandex.ru) under the voucher number 40. A genomic DNA is deposited at National Research Center ‘Kurchatov Institute’ (http://eng.nrcki.ru/, contact person: Svetlana Tsygankova – e-mail: svetlana.tsygankova@gmail.com) under the voucher number PP1C. The data that support the findings of this study are openly available in GenBank of NCBI at https://www.ncbi.nlm.nih.gov, reference number MZ065355. The associated BioProject, SRA, and sample numbers are PRJNA742656, SRR15000149, and SAMN19967399, respectively.
